# An assessment of khat consumption habit and its linkage to household economies and work culture: The case of Harar city

**DOI:** 10.1371/journal.pone.0224606

**Published:** 2019-11-05

**Authors:** Zerihun Girma Gudata, Logan Cochrane, Gutema Imana

**Affiliations:** 1 Haramaya University, CHAMPS Ethiopia, Harar, Ethiopia; 2 Institute of Policy and Development Research, Hawassa University, Hawasa, Ethiopia; 3 Haramaya University, Department of Sociology, Harar, Ethiopia; Technical University of Kosice, SLOVAKIA

## Abstract

**Background:**

This study investigates khat consumption habits and its linkage to the economy of a household and works culture in its ‘homeland,’ Harar. Khat consumption habit includes practices that are performed before, during, and after consuming khat. In Harar, it has permeated the local economy, social, political and spiritual lives. To evaluate how khat consumption habit is related to the economy of a household, this study compares the living standard and characteristics of khat consumers and non-consumers.

**Methods:**

Both qualitative and quantitative approaches were used. Cluster sampling and lottery methods were used to identify respondents. Data were gathered through individual interviews and non-participant observation.

**Results:**

The results of the study indicate that khat consumption habit affects the economy of the consumer household by negatively influencing their income usage and time management. Consumer households have significant, additional burdens on their income and time. The average monthly expenditure of a household on khat ceremonies is 1,800 ETB (30% of their income) and consumers spend an average of 112.5 hours monthly on khat related activities only. The habit of khat consumption also negatively associated with the work culture of consumers, as they leave for lunch break early and come back to work late.

**Conclusion:**

Khat consumption habit does have a linkage to the bad economic situation of consumer households. It places a significant financial and time burdens on individuals, and as a result society. The work and saving culture of khat consumers are negatively affected. Khat consumption forces many into a cycle of borrowing and indebtedness. Concerned bodies should not underestimate the impacts of khat consumption on individuals and society.

## Introduction

*Khat*, *chat* or *qat* (Latin: Catha edulis; hereafter khat) is a chewable green leaf that has a euphoric effect on the consumer. Consecutive use potentially leads to addiction. In Ethiopia, one of the East African nations, khat production, trade, and consumption has been long practiced, especially in Harar [1, 2]. Richard Burton, who traveled in the region in the 19^th^ century, in his *First Footsteps in East Africa*, discussed the khat trade around Zeyla and Barbara and its popularity in Yemen and parts of Arabia [3]. Moreover, Burton indicated that it was Shaikh Ibrahim Abu Zaharbui who introduced khat into Yemen from Abyssinia (Ethiopia) [3]. According to Ezekiel [1], the use of khat, the making khat drinks, and chewing khat leaves may have first started with medical purposes. Khat has recently emerged as one of the most controversial products in Eastern Africa and the Middle East due to its economic, social, cultural and political impacts [2, 4]. In Ethiopia, the cultivation, production, and consumption of khat are expanding at an alarming rate [5]. Although there is an emerging body of literature on khat (e.g. [6, 7, 8, 9, 10, 11, 12]), research remains quite limited. Available literature focuses largely on the consumption trends of university students and on biomedical aspects.

This paper assesses khat consumption habits and its linkage to the economy of the household and work culture, specifically within Harar, in eastern Ethiopia. Historically, Harar was known as the epicenter or homeland of khat in Ethiopia [13], which is why this study has focused upon it. There are several studies that discuss khat consumption as a habit, tradition, and culture (e.g. [4, 14, 15]). Some have discussed khat culture, consumption habits, and tradition by relating it to both the production and consumption of khat [1, 2, 4, 15]. Ezekiel equates khat culture with *Barcha*, a situation where “users congregate in a designated room in private houses and lie on their sides on a pile of cushions to meditate, read and engage in the talk of the town” [1]. While consuming khat, individuals drink water or tea to reduce dryness of the mouth, take a pinch of sugar or sip soft drinks to moderate the bitter taste and may smoke cigarettes as they chew [1]. Although the above variables are some of the important points considered in this study, khat consumption is also related to the cultural or ceremonial practices before, during, and after a session wherein khat is chewed or consumed. For this study, the focus is upon regular khat consumption, as opposed to occasional or irregular consumption. To be a habit, it must be consumed regularly for a long period of time.

The basic difference between khat consumption and habitual khat consumption is the frequency and integration of khat chewing into the life of an individual or community. In Harar, almost all of the people consume khat, however not all these populations have the habit of khat consumption. For instance, some people only consume khat on the weekend, once every two weeks or once a month, or at special events such as wedding and funeral ceremonies. According to this study, this is not a habit, these people only consume khat for entertainment and only ‘when khat finds them’, as explained by study participants. In defining and applying the concept of khat consumption habit in this study, the idea of repetition and continuity is emphasized. It is a state in which a person uses khat at least two or three times a week and feels uncomfortable, bored and displays unusual behavior, including “depressive mood, irritability, anorexia, and difficulty to sleep” and mood swings [16,17] when not consuming or finding khat.

Harar is thought to be the origin of khat and the main historical location for its consumption, a practice that is claimed to have deep historical roots [1, 2, 13]. For regular consumers, khat consumption habit involves chewing at one or more of the ‘sessions’ of chewing in a day: starting with *ijabana (eye-opener)* in the morning, followed by *barcha* in the early afternoon, and finally the *atoorara* session in the evening. The latter of these sessions is particularly common during special events or times of the year, such as during Ramadan, during which neighbors or relatives congregate and consume khat together. In some parts of Ethiopia, khat consumption habits also include associated activities, such as *chabsi*, a drinking time, however, this is less common in Harar. A societal khat consumption habit is indicated by the frequency of chewing, expenditure, and the integration of khat into the life of the society and its normalization throughout the culture, economy, political and spiritual realm of life.

The issue of khat is complex and multidimensional. It is not possible to cover all aspects of it in a single study. This paper contributes evidence to a broader discourse on the relationship between khat consumption and household economy. Advancing knowledge on the diverse impacts of khat is important, as decision-makers grapple with how to develop appropriate policies and regulations. Governments and policymakers face significant dilemmas when seeking to engage with khat. According to Cochrane and Girma, these decision-makers are faced with a ‘difficult, and at times impossible, task’ of considering a myriad of political, economic, social and cultural components [8]. The current state of affairs is one of ‘quasilegality’ of khat consumption, with laws that are vague, flexible, susceptible to manipulation and lie between lawlessness and complete legality [18].

The available research on khat tends to provide polarized perspectives. There is a debate about whether khat consumption habit has a negative or positive impact on the economy as well as if khat is a barrier or facilitator to development and poverty alleviation in the country [19]. On the one hand, there is a group of scholars (e.g. [13, 20, 21]) who hold that khat chewing leads to a loss of work hours, divorce, decreased economic productivity, malnutrition, and diversion of money in order to buy further khat. This group also indicates that khat is linked to absenteeism and unemployment, which may, in turn, result in a decline in overall national economic productivity. This group of scholars tends to also highlight the negative health impacts of khat [21, 22, 23, 24]. On the other hand, a second group of scholars (e.g. [10, 12, 25, 26, 27]) hold that khat has positive outcomes, such as increasing peaceful social coexistence, improving social connectivity and even health, promoting productivity, and when consumed “moderately”, improving performance and increasing work output because it stimulates and postpones fatigue. Consequently, working hours, and possibly productivity, are suggested to decrease when khat is not used because of reduced motivation. With regard to khat cultivation and production, this group of scholars points out that khat cultivation doubles the income of farmers and boosts their income and general living standards [1, 25, 28, 29].

This study argues that khat consumption habits can negatively associated with the consumer family’s economy and time management. Although there is no such difference between consumers and non-consumer households on their monthly income, great variances were found on how they use it. The former has an additional burden related to khat chewing ceremony. Consumers not only spent great amount of money on khat but also time. Khat consumption habit also complicates the work culture of chewer households. Consumers leave work early and come back late to work. We recognize that not all aspects of khat have been researched in this study, but we aim to contribute evidence to a debate that has often been value-based.

## Methodology

### Ethical clearance

Ethical clearance was obtained from the Postgraduate Program Directorate of Haramaya University before conducting the study, it states: The Postgraduate Program Directorate of Haramaya University has made a thorough review of this study, conducted by Mr. Zerihun Girma, Prof. Logan Cochrane, and Dr. Gutama Imana before it was implemented following the international, national, and university ethical clearance guidelines. Hence, the research plan (proposal) has met the requirements of the National Statement on Ethical Conduct in Human Research (2007), and full ethical approval has been granted for the conduct of the research, which also accomplished as per the approved conditions. None of the investigators participated in the decision making and voting procedure. Therefore, the postgraduate program would like to ensure any concerned body that the work was conducted as per the requirements for research involving humans as a subject and that it has fully implemented all the human handling during the undertaking of the work.

### Research methods

This study used both qualitative and quantitative research approaches to assess the linkage between khat consumption habits and economy of households and work culture. The study applied a survey method by selecting households, through sampling, with the objective of making inferences about the study population. The population of the study consisted of the residents of Harar city and the sampling consisted of households in the city. A cluster sampling method was used to identify the respondents. Households in blocks or clusters of residents of Harar city were identified using *Google Earth* and maps from the Harari Regional State Tourism Bureau. Using the map, households were divided into 61 clusters or blocks, guided largely by transportation infrastructure and settlement patterns. Letters were assigned for each block. Following the classification, a lottery method was used to select clusters from where the data would be collected. The selection of districts using a lottery method continued until the required sample size was achieved. In total, six clusters were included in the study.

The sample size was identified using Slovin’s formula, n = $\frac{N}{\left(1+Ne^{2}\right)}$ where ‘n’ is the number of samples; ‘N’ is total population and ‘e’ is a margin of error.

According to the most recent national survey, the number of households in Harar city is 28,322 [30]. Using a confidence level of 95 percent, with a margin error of 0.05, a 398 sample size was identified as the respondent population or number of households. Next, the sample size was divided into two groups, 201 consumers (chewer) and 197 non-consumer (non-chewer) households. The percentages and means in the study were calculated separately for each group. Whereas a ‘consumer household’ was defined as a household where the family head (in most cases the husband) and other family members engage in khat consumption habitually, and a ‘non-consumer household’ was defined as a household where the family head (mostly the husband) does not chew khat, although some other household members may. There are some limitations with these definitions, such as how we generalize the consumer and non-consumer household. The focus of this study is upon the economic influence of household members who engage in khat consumption, and specifically, the role played by the household head as this has an important economic impact on the other members of the household. In this study, a ‘household head’ refers to anyone in the household who is primarily responsible to make decisions on behalf of the family. A ‘household’ is composed of family members that reside in one house or apartment. These family members are economically interdependent and affect one another. This may include a husband, wife, children, and relatives. Due to population and housing patterns typical in this region, one dwelling may include more than one ‘household’.

In addition to household surveying, 20 key informant participants were purposefully selected from the khat consumer households. The qualitative data that was collected during this process provided additional insight into revealing a more contextualized picture of the relationship between khat consumption and household economy. These interviews also included other household members, in addition to those engaging in khat consumption. In some households, where the khat consumer tried to significantly underestimate the impact of consumption in their family and tried to focus only on the ‘good’ side of khat, the non-consumer family members played a significant role in revealing the real effect of khat on their family.

### Methods and instrument of data collection

The following data instruments were used in this study: A structured self-administered *(1) interview guide* was used for data collection. As the researcher moved from house to house, it offered a good opportunity to (2) *observe* the environment of both khat consumers and non-consumers and their households. A checklist of who and what to observe was prepared to make the observation consistent throughout all data collection. Lastly, (3) *key informant interviews* were also conducted. The interviews mainly focused on the non-consumers who live with khat consumers to better understand the effects of khat from different perspectives.

After identifying blocks using a lottery method, household selection and interviewing household heads started. However, in some areas (like *Jugal*) it was difficult to find non-consumer households, and as a result, sometimes it was necessary to jump over some households in search of non-consumer households. In addition, it was challenging to get economic information from some families. Some household heads did not know their exact income (largely due to irregularity) and others were reluctant to share it. Since the focus of this study is to assess the linkage between khat consumption habits and the household economy, it did not consider gender differences and the differential impact of khat consumption. The gender-disaggregated data on consumption and the gendered nature of khat consumption and its impacts is an area that requires further study.

In the interview guide, questions were designed in such a way to gauge the response of respondents regarding proposed statements /situations as well as to assess the effect of certain features of khat consumption habit on their household. Determining an appropriate time for the interviews was an important consideration in this study, particularly for interviewing khat consumers as this could greatly influence the answers provided regarding some questions. Data collection was conducted with those who engaged in khat consumption before they began chewing; and before chewers who could not find khat felt the negative effects of not consuming. Most of the available research on khat neglects to consider the importance of data collection timing. Studies indicate that khat can create a ‘feeling of well-being, increased level of energy, mental alertness and self-esteem, sensations of elation and excitement, and enhanced imaginative ability . . . .’ [31, 32]. These emotions and feelings can influence the information provided, particularly when questions relate to self-reported data and perceptions and therefore the quality of the data. In recognition of this, the current study seriously took into consideration the issue of timing when interviewing khat consumers. Non-consumers who live with consumers were interviewed separately so as to ensure they were able to freely speak about the effects of khat consumption on their families. While asking questions, the environment of the household, its quality, and available home materials were observed.

## Results and discussion

### The study participant and their khat consumption habit

Before discussing khat consumption and economy of a household it is important to look at some profile of the study participant and their consumption habits. This study focuses on a household by classifying into two groups: consumer and non-consumer households. The former exhibit the traits of khat consumption habit while the latter not. From the participants in the interview schedule, 201 were consumer households with the habit of khat consumption and 197 were non-consumer households. From the randomly selected respondents (household heads) who consume khat, 75 (37.3%) were between the ages of 19 and 29, 69 (34%) were between the ages 30 and 39 and only a small number were above 50. More than 70% of the consumers were between the ages of 19 and 39 (S1 Table). This age set can be considered as the working-age population, an energetic and more productive segment of society. Most of the households included in this study were headed by a male husband. However, there were some houses headed by females, this was common where women are single, widowed and divorced. A few households were headed by an older son or relatives, typically where the husband was absent.

When we look at the educational status of the respondents: most of the khat consumer household heads, 93 (46.3%), were university graduates, which was slightly a higher rate when compared to non-consumers (72; 36.5%). This indicates that not only young and productive individuals consume khat, but also the educated members of society. The higher level of consumption amongst educated individuals aligns with the findings of Yeshigeta and Abraham, who indicated that a large portion of Jimma University staff consumes khat [33].

Regarding work, participants were divided into three groups: employed, underemployed and unemployed. Employed refers to having one’s own business or a job that pays wages or a salary. Underemployed means employed part-time. Unemployed indicates not employed by any private or public organizations or offices and earns no money. From the study participants, 102 (50.7%) consumers and 141 (71.6%) non-consumers were employed as full-time workers. Whereas, 54 (26.9%) consumers and 36 (18.3%) non-consumers were underemployed, and 45 (22.4%) consumers and 20 (10.2%) non-consumers were unemployed (S3 Table). This means that a higher number of consumers in the study were underemployed or unemployed compared to non-consumers. It is not clear whether khat consumption causes unemployment, or if unemployment causes khat chewing. However, when compared with non-consumers there is a relation between khat consumption habit and employment status. In Harar, there are many opportunities that enable khat consumption even if someone does not have money or a job. One example is the high degree of social cohesion among the people: if you do not have the money you can easily join others and consume khat with them. Another is the relatively low price of khat compared to the price of khat in other cities like Jigjiga and Dire Dawa, which makes khat easier to obtain. Additionally, khat is readily available due to the presence of large khat markets, where one can easily beg for khat and take *garaba* (leftovers).

In almost 90% of the consumer households, it is either the father, mother and father, or the whole family who consume khat. This shows that, in Harar, in most instances it is the influential members of the family who consume khat (e.g. head of household, parents). In most of the consumer households, it is the father who chews khat, as was the case in 113 households (56.7%). This has implications on the household’s overall socio-economic wellbeing. As with other parts of Ethiopia, it is the male household head who makes most of the decisions. Moreover, in most instances it is the husband who earns income for the family. Key informants also indicated that these male household heads can use their monopoly on household income to fulfill their need for khat at the expense of basic family needs. In addition, 31 (15.4%) of the households responded that the whole family consumes khat and 36 (17.9%) responded that both the mother and father use khat (S2 Table). Key informant participants indicated that there is greater tolerance with regard to khat consumption in the family where the mother and father or the whole family consume khat than in households where only father or only some members consume khat.

From those who participated in the interview schedule, 60 (29.9%) had consumed khat for more than 15 years. Another 48 (23.9%) had done so for 10 to 15 years (S2 Table). In general, most had consumed khat for more than 5 years, indicating that this is not a new socio-cultural trend, rather an already established and accepted practice. What the authors believe is that the longer duration of consumption has implications for linkages to habitual consumption, however further study is required to assess dependency rates. The longer-term use, meaning greater than five years, suggests that khat consumption has become intertwined in socio-economic lives and therefore poses difficulties if initiatives were put in place to alter the practice. In Harar, exposure to khat culture starts in early childhood and continues to old age. Children grow up not only seeing khat but also touching, tasting and feeling it, unlike in other parts of Ethiopia where they learn from their peers after they reach adolescence. Studies in other parts of the country indicate that most youth learn about khat in university. For instance, in Jimma, 40% of consumers started doing so in universities [33]. In Harar, however, khat consumption is learned from an early within the family and household.

In contrast to commonly held perceptions (held by both scholars and the public) that the people of Harar consume khat for religious purposes (e.g. [26, 34]), particularly for prayer, none of the respondents mentioned that they consumed khat for religious purposes. Rather, 129 (64.2%) of the respondents self-reported that they consume khat to get energy for work and 48 (23.9%) responded that they use khat for entertainment. Aligned with the purposes for consuming khat, 96 (47.8%) and 78 (38.8%) responded that they could not work well or be happy if they did not consume khat, respectively (S2 Table). This implies that unless those who have khat consumption habit consume khat they believe that they cannot work effectively or properly. This is an alarming development since khat production and consumption is expanding in Ethiopia [5].

The majority of consumers (65%) chew at least once a day; 99 (49.3%) self-reported that they consume once a day, 23 (11.4%) two times a day, 6 (2.9%) three times a day, and 4 (1.9%) throughout the whole day (S2 Table). Similar to the idea of khat consumption being linked to religion, this finding differs from the ideas of Ezekiel, who wrote that khat was consumed three times a day [34]. In this study, only 6 (2.9%) people reported that they consume khat three times a day. Moreover, it was found that these six respondents are farmers from the surrounding rural villages who own khat farms and live in Harar city. The impacts of khat consumption habits on a household such as these are not clearly visible because expenditure on khat is zero or very low, and conflict between consumers and non-consumers in these households was also absent since the whole family consumed or at least accepted khat consumption. Although further study is required to understand this specific household type, the findings indicate that there are differences between the rural khat consumers that Ezekiel focused on and the urban consumers that this study focused on, as well as households that cultivate khat.

Most of the political leaders in Harar city also support khat production and consumption. They stress the significance of khat for the farmers in the surrounding villages and the income it generates for the country. The chairperson of Harari Regional State equates the significance of khat for its people with oil in the Arab states (during the study we met with the chairperson of Harari Regional State Council). When asked about the urban consumers, the chairperson strongly argued, “the problem is with the chewers not with the khat itself.” He inquired if we, as researchers, were ready to include the ‘good side’ of khat in the study. Undoubtedly, there are people who benefit from khat; farmers who cultivate it obtain a higher sale price than other potential crops, multiple levels of government obtain significant tax revenues and the broader economic benefits from the vibrant khat trade. Like the chairperson, most of the scholars who defend khat base their premises on the positive impact khat cultivation has for rural farmers and the economic impact gained by khat traders (e.g. [4, 26, 34]). National revenues from the export of khat are significant [5], however sub-national data on taxation is limited.

To see the study participants’ habit of khat consumption in a more detail way we have also asked, how long they could stay if it is difficult to find khat or there is insufficient money to buy. For this line of questioning, 66 (32.8%) and 45 (22.4%) of the respondents revealed that they could only stay for two or three days without consuming khat ‘normally’ or without showing the need for khat, respectively. The other 57 (28.4%) asserted that they can stay up to a week (S2 Table). However, the facial expressions on some of the participants suggested that they were unhappy with even considering such a situation. Some respondents quickly questioned the appropriateness of the question, as it had never come to their mind or that they had never imagined staying some days without khat. However, most of the consumer participants tried to explain that they were not dependent on khat.

Based on the data from the key informant interviews and observation, one could classify two types of consumers in this study: (1) those who say, ‘life for me is chewing’ and (2) those who say, ‘I only chew sometimes’ (informally, the former are called *jazba* and the latter *chawa*, however, these words are offensive for some consumers). These divisions are mostly based on lifestyle and khat consumption practices. The former always consumes khat, khat consumption is involved in their day to day activities, and without it they cannot do anything or feel depressed. From consumers who were involved in this study, there was a considerable number who could be grouped into this category (as this is based on qualitative data, we do not provide a figure; further study is required to assess rates of dependence). This group of consumers practices *tarzina*, which is the practice of holding a large amount of khat in one side of their mouth for long periods of time to extract juices. This is done everywhere; while working, walking on the street and while doing business. Individuals in this category do not hide their khat consumption. This group is mostly unemployed, day laborers or underemployed. On the other hand, those who say ‘I only chew sometimes’ are secretive about their consumption habits. These consumers do not want to be called *kami* or ‘chewer’. They do not consume in public and, although they too do *tarzina* while chewing, they do not do so in public. During the interviews, individuals from this category would frequently say, ‘I only chew sometimes, two or three times a week.’ After consuming they clean their teeth and mouth to hide their consumption from others. As a result, they are cleaner and more protective of their hygiene. Most of the individuals from the latter group are employed as officials or government workers. Nevertheless, both groups exhibit the traits that indicate habitual khat consumption, although the degree differs.

### Khat consumption habit, income, and expenditure

The interview participants were asked to provide the average monthly income of their household, the average daily expenditure of their family and their saving practices. Regarding this, there were a lot of challenges during the data collection process. First, many individuals did not know their exact monthly income because it was not consistent. Similarly, expenditure varies. In both cases, an approximate income figure and a minimum average expenditure of the household were sought. Second, some of the respondents were reluctant to provide financial information, particularly their saving capacity. Thus, it was necessary to provide participants with additional information about the objectives of the study to ensure it was understood that the data would not identify individuals and focused on societal trends. While all efforts were made, these figures are best viewed as general trends, rather than specific data points on income and expenditure.

The result shows that there is no great difference regarding the monthly income of consumer and non-consumer households. There is a slight difference only on the high-income level, but almost no difference among many low-income consumer and non-consumer households (S3 Table). Consumers have a slightly better education, as indicated above, but this was not reflected in their income. Although the actual reason for this needs further study, previously 48% of chewers indicated that they do not work effectively if they do not consume khat, thus, the difficulty to find khat might contribute to this.

**Fig 1:**
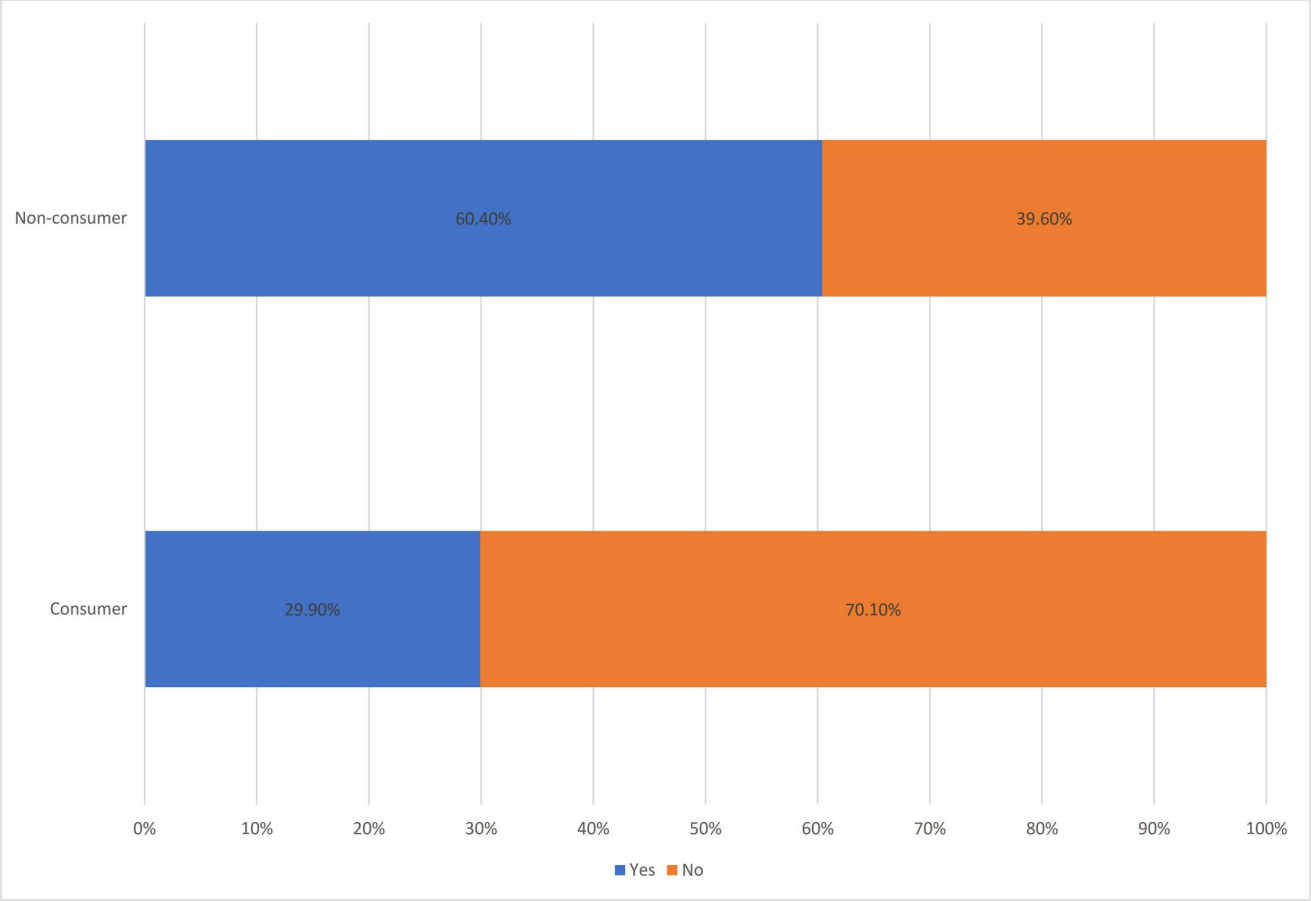
Ownership of bank account, consumer vs non-consumer household.

One key difference between consumer and non-consumer respondents was with regard to how they used their monthly income. For instance, while only 60 (29.9%) consumers had bank accounts, 119 (60.4%) non-consumers did (Fig 1). Although owning bank accounts is only one manifestation of saving practices, this indicates the presence of the intention or plan to save their income. In addition, although having a bank account is a requirement for some activities, this is true for both consumer and non-consumer households. For instance, government officials are required to have a bank account, since their salary is deposited directly. Nevertheless, the difference between consumer and non-consumer respondents about being employed and having a bank account is vast (S3 Table and Fig 1). The researchers attempted to clearly convey the objective of this question in the interview process, which was not only to see whether they had a bank account or not but to assess their saving practices and to see whether they have intended to save their income or not. Because of this, there were government employees who had bank accounts but responded ‘no’ to this question as the account was only used to withdraw salary. The findings demonstrate a difference between consumers and non-consumers regarding their saving practices and saving culture.

In addition, there were many discussions during the interviews regarding the lack of saving practices among khat consumers. There were households where wives interjected and commented on the lack of saving practices. In one of our interviews, for instance, one housewife said, “as long as there is khat, there is no saving in this house.” In sum, the data shows a narrow difference between consumers and non-consumers in terms of their monthly income but a significant difference with regard to how they use their income. The following sub-section provides two more reasons why consumers have less overall savings and/or face difficulty saving their income: first, there is always a shortage of money at the end of the month and second, the additional expenditure on khat.

Regular consumption of khat requires a lot of time and money. In addition to basic family expenses, engaging in khat consumption creates additional burdens on the families. Although the price of khat fluctuates significantly, we tried to gather average data on how much money do consumers spent on a single chewing session. The price difference is not only between seasons, in which the price of khat drops in summer (*kiremt*, the rainy season in Ethiopia) and rises in winter (*bega*, dry season in Ethiopia), but also between hours of the day. During the data collection, we able to observe that the price of 300–500 grams (one pack, for one consumption session) of high-quality khat from Awaday market is 200 ETB (1USD = 28.84ETB) around 2:00 PM and drops to 150 ETB after 4:00 PM (during the dry season this can reach 500 ETB), this was low as it was the rainy season. Given the fluctuating price of khat, consumers were asked to give the minimum average expenditure for one consumption session.

The result shows that 51% of consumers spent greater than 50 ETB per session. The mean of total expenditure on one khat consumption ceremony was 75.8 ETB. This is a large expenditure relative to their income and expenses for basic family needs (S3 and S4 Tables). The mean expenditure by khat consumers for basic family needs is 52 ETB. Those who spend more than 150 ETB for one khat session have higher incomes per month and consume only two or three times per week, who accounted for 22.4% of the consumers. On the other hand, those who spend more than 150 ETB per day on basic family needs (like food and drink) only account 11.9% of the consumer households, while those who spend 50 to 150 ETB per day for basic family needs account for 34.3% (S3 and S4 Tables). This reveals that khat claims a greater share of the expenditure in consumer households compared to the expenditure for the basic needs of a family. To illustrate, consider one family in Harar wherein a government worker earns 6,000 ETB per month. The individual consumes khat that costs 150 ETB per session, three times a week. Their monthly expenditure for khat is 1,800 ETB (30% of their income).

**Fig 2:**
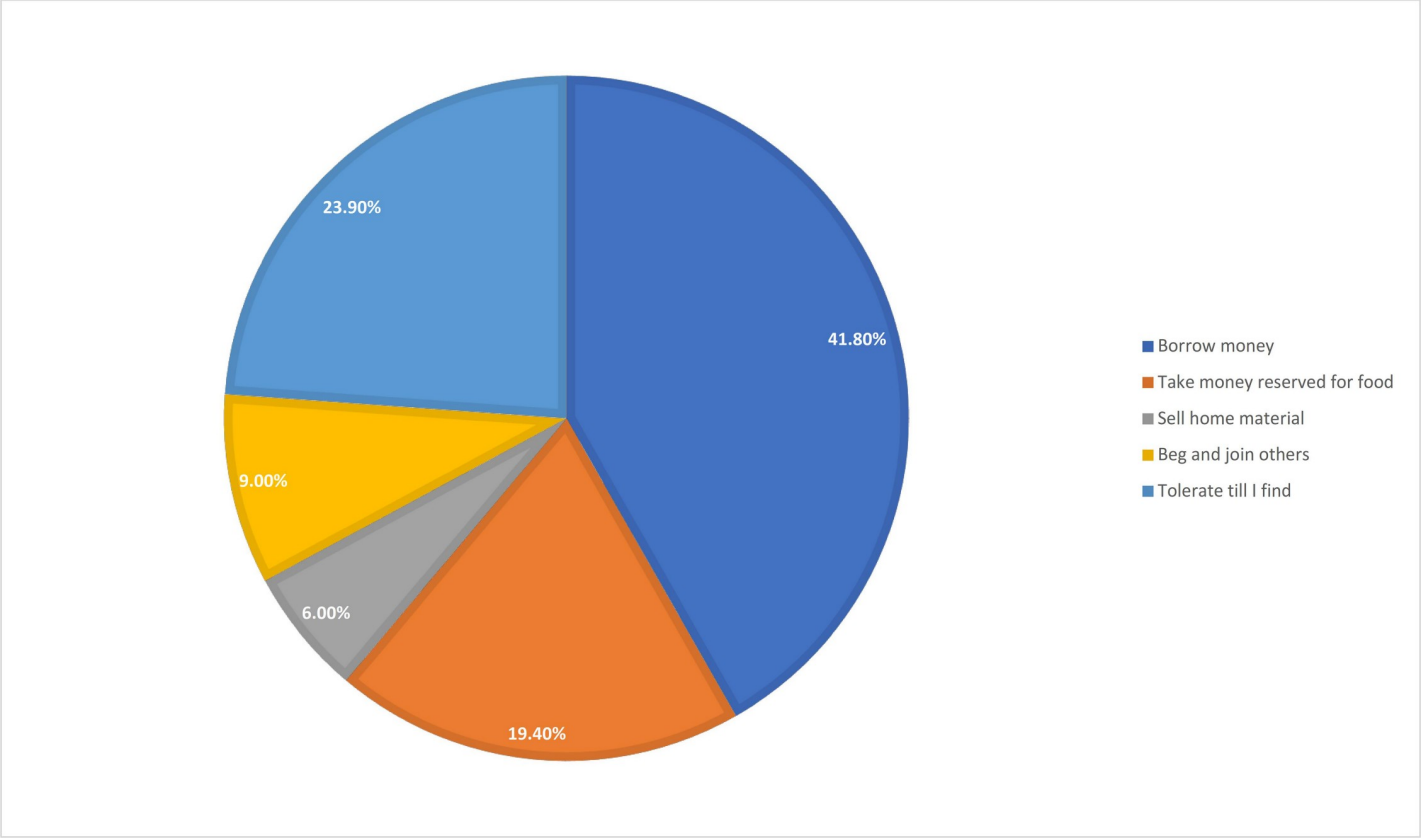
Measures taken by consumers if they do not find money to buy khat.

We have also asked ‘what would you do if you need khat badly, but you do not have money’, 84 (41.8%) consumers responded that they would borrow money, 39 (19.4%) said that they would use money reserved for food and 12 (6%) responded that they would sell home materials to satisfy their need for khat (Fig 2). On the other hand, only 48 (23.9%) responded that they would tolerate the condition and wait until they find khat. The rest 18 (9%) responded they would seek khat from others.

Non-consumers, who had a consumer family member, who participated in the key informant interview indicated that borrowing money for khat costs a lot for their family as it leads to a continuous cycle of borrowing, interest payments, and indebtedness. There are consumer household heads who obtained a monthly salary and first had to pay off their debts when they got paid. Since the debt is paid from their salary, the remaining money may not be enough for the expenses of the month, which in turn forces them to borrow money again. For some families, this is an unending cycle of borrowing and indebtedness in which one becomes trapped. Wives who have consumer husbands disclosed that the greatest negative effect of khat consumption for the household is taking money reserved for food and/or selling home materials to buy khat. This leaves the whole family, including non-consumer members and children, with reduced food and home materials. While the researchers moved from house to house it was common to see consumer households in such a situation, particularly those who could be classified as a person who states, ‘life for me is chewing’. Individuals from this group run out of home materials after selling them to secondhand material collectors at a very low price. Most of these consumers do not have mobile phones because it had been sold in order to purchase khat. This causes family quarrels and conflicts, which can easily lead to family breakdown and separation or divorce, which was the case for some participants of this study.

Another problem related to khat consumption is drinking alcohol, however, in Harar, this is not as such connected with khat consumption. Only 21 (10%) of the participants believe that drinking is essential after chewing, which is known as *chabsi*. Forty-five (22.4%) replied that drinking is somewhat desirable, however, the great majority, 135 (67.2%), believed that drinking is not necessary after *barcha*, the khat chewing ceremony. In this study, alcohol drinking adds more challenges and costs for 66 (32%) of consumer households. The same is true concerning the use of other substances, as 87 (43.3%) of the consumers use other substances like tobacco and *shisha* in addition to khat (S4 Table).

### Khat consumption habit, time management, and work culture

**Fig 3:**
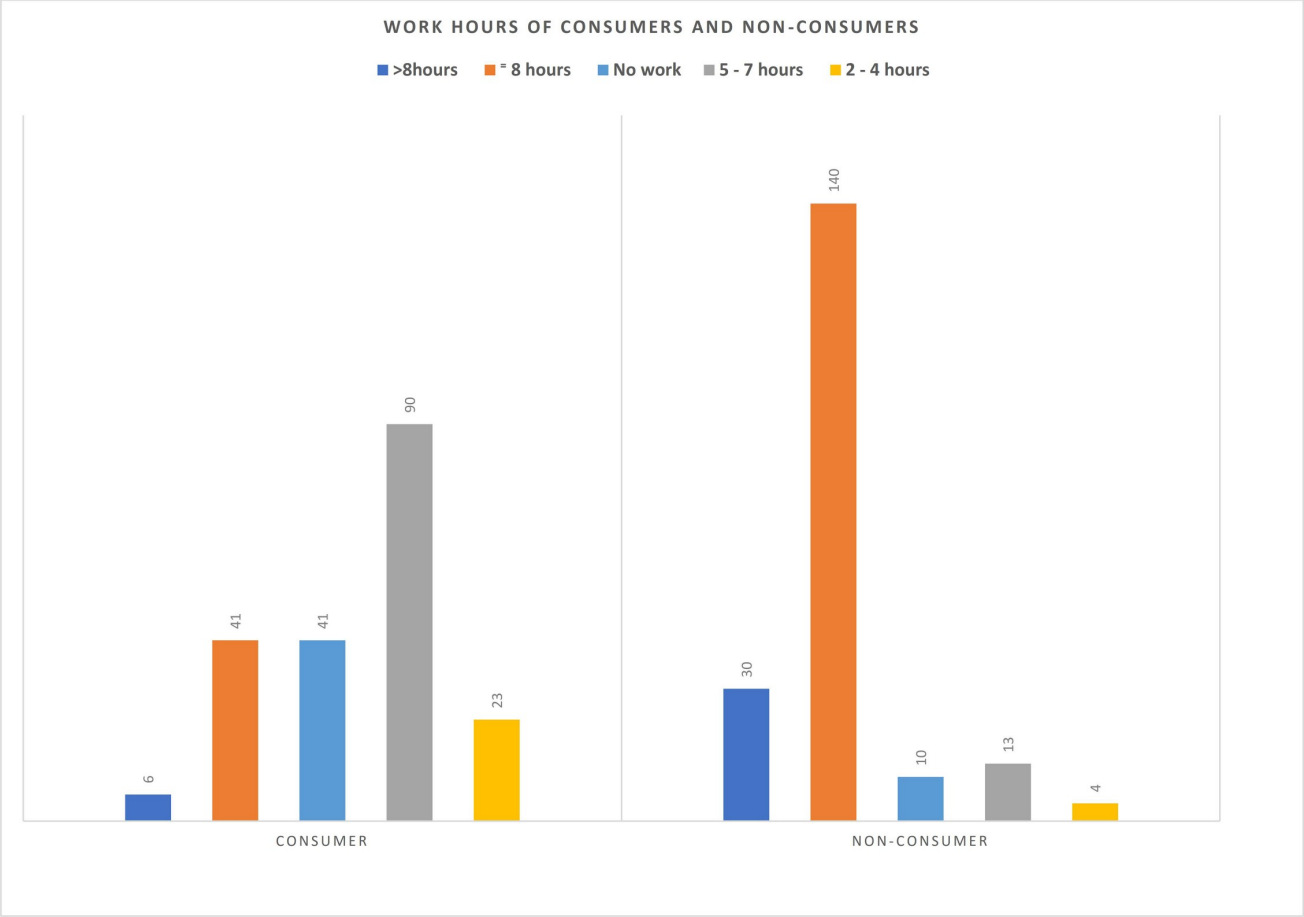
Work hours of consumers and non-consumers.

One way to assess the linkage between khat consumption habits and work culture is to examine hours spent at work. As noted at the outset, some argue that khat enables improved productivity and efficiency, while others argue it negatively impacts work habits. Fig 3 presents the difference between consumers and non-consumers regarding work hours per day. It was found that 41 (20.4%) consumers and 140 (71.1%) non-consumers responded that they work 8 hours per day. The majority (90; 44.8%) of khat consumers responded that they work between 5–7 hours per day (while this was 13 or 6.6% for non-consumers) and 23 (11.4%) consumers responded that they work between 2–4 hours per day (this was only 4 or 2% for non-consumers). The mean working hours for consumers was 6.5 hours per day while it was 8 hours for the non-consumers. These results indicate that consumers work less than the standard hours of the working day, which is directly linked to khat consumption (discussed below).

If they work less how do consumers spent their time? In addition to expenditure on a single khat chewing session, we have also asked for an average time spent on a single khat chewing session. Of the participants, 36 (17.9%) of the consumers spent greater than 5 hours on one chewing session. The great majority, around 123 (61.2%) spent 3–5 hours and 42 (20.9%) spend 1–2 hours on a single khat consumption session (S4 Table). This means that consumers spend an average of 3.75 hours consuming khat and/or with khat related activities. Thus, on average, chewers spent 112.5 hours monthly on khat chewing only.

**Fig 4:**
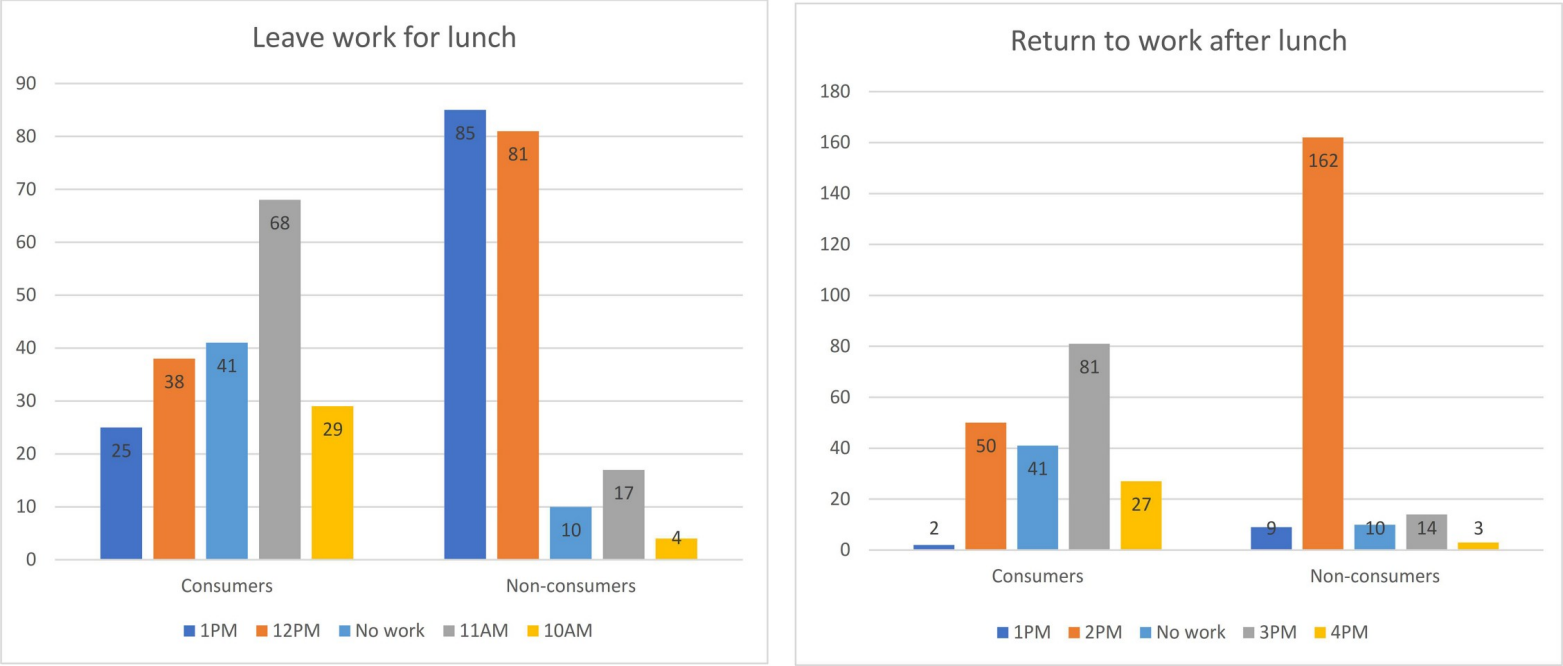
Time consumers and non-consumers leave and return to work after lunch.

Moreover, both consumers and non-consumers were asked to provide an estimated average time at which they leave work for lunch and return to work after lunch. Here, ‘work’ does not necessarily denote government work, it can indicate any gainful activity that is private or public, full time or part-time, formal or informal. Fig 4 presents a comparison between consumers and non-consumers regarding working hours. The results show that 85 (43.1%) non-consumers and 25 (12.4%) consumers leave work for lunch at 1:00 PM whereas 81 (41.1%) non-consumers and only 38 (18.9%) consumers leave work at 12:00 PM (the official). On the other hand, while 29 (14.4%) consumers leave work at 10:00 AM, only 4 (2%) non-consumers did so. The calculated mean of leaving work for lunch is 11:20 AM for consumers and 12:20 PM for non-consumers. When it comes to returning to work after lunch in the afternoon the reverse is true. Most of the government officials in Harar return to work from 1:30 PM to 2:00 PM, but only 50 (24.9%) consumers did this, whereas 162 (82.2%) non-consumers did. Surprisingly, 81 (40.3%) of the consumers return to work at 3:00 PM, which was the average return hour after lunch for chewers (Fig 4). That means, most of the consumers return to work in the afternoon only to work for two and a half hours.

**Fig 5:**
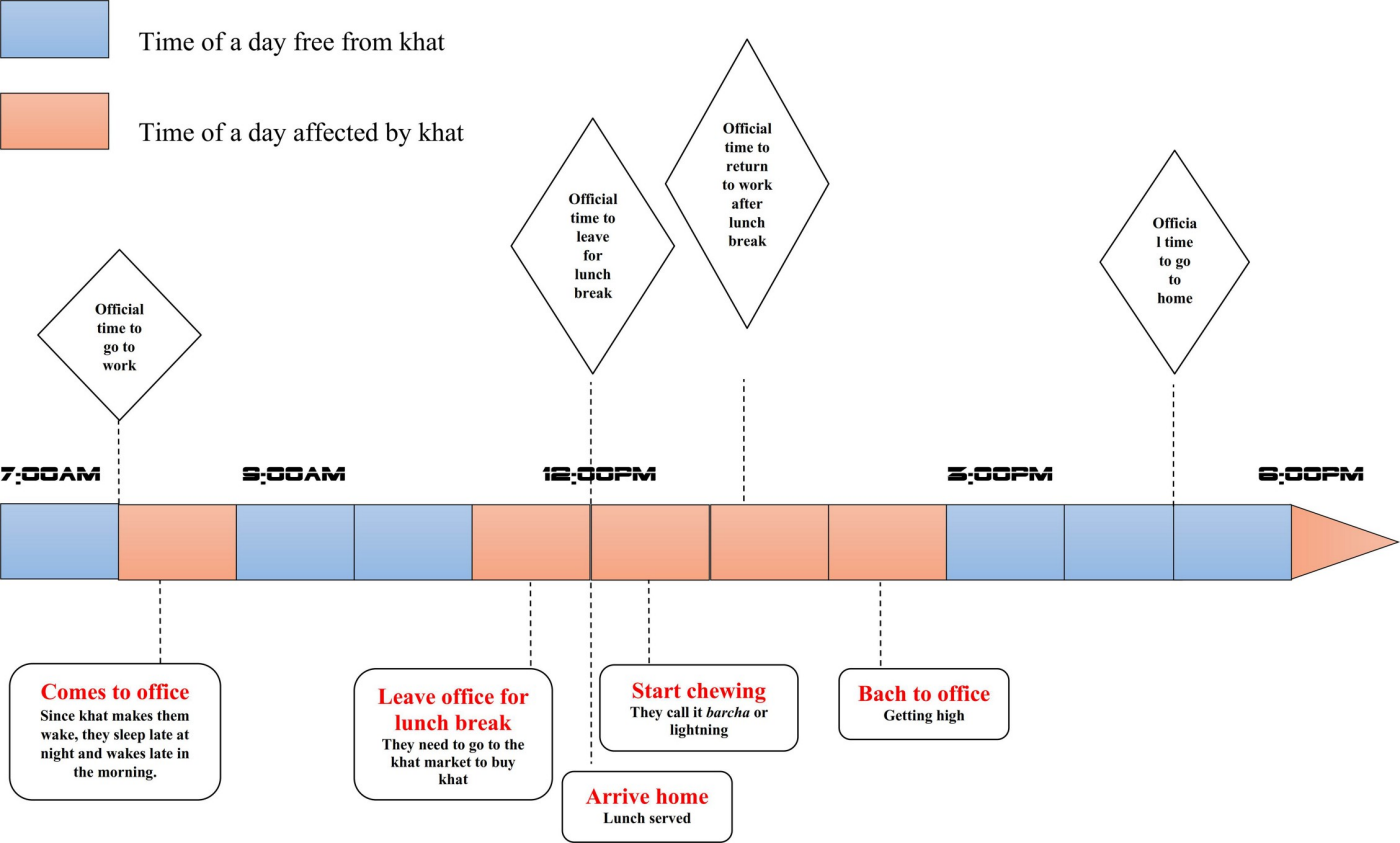
Timeline of consumers in a day.

These findings reveal that the majority of consumers leave work for lunch early and return to work late. The impact of this is not only on the economy of a household, but also work discipline, punctuality, honesty, service for customers, and the overall integrity of an institution. For instance, one of the consumer participants, who work in a government office, explained that ‘if I get home for lunch at 12:00 pm or before, the lunch will be served at around 1:00 pm. Then, I need to sit and chew khat at least for 3 hours to get ready for the afternoon work’ (see the aggregated timeline of consumers in Fig 5). The same finding was presented in a study from Jimma University by Yeshigeta and Abraham [33]. They indicated that around 50.4% of khat consuming staff have one or more times missed their regular work at the university because of khat consumption and that 54.5% of consumers come late after consuming khat or leave work early to consume khat. Yeshigeta and Abraham concluded that khat has a negative impact on service delivery and the teaching-learning process [33].

The timeline (Fig 5) presents hours of a day affected by khat consumption practices. The information was obtained from consumers and non-consumers who participated in the key informat interview and aggregated. The times in red indicate the negatively affected times of the workday. For instance, in the morning from 8:00 am to 9:00 am, some consumers go to work late. Often, this is because those who consume at night stay awake late and as a result do not wake up early. In midday, 11:00 am to 1:00 pm is negatively affected as consumers start to show some signs of the need for khat such as yawing, stretching and getting bored. They report facing difficulty concentrating. This urge pushes many consumers to leave work by 11:30 am. Before going home, they go to a khat market to buy khat. By 12:00 noon they arrive home, and lunch is served, after which they begin to consume khat. The consumption may start at 12:30 pm and for most chewers lasts up until 2:30 pm. This is called *barcha* or ‘lightning’, because of its necessity to speed or rush to go back to work. The time periods that follow, from 2:30 pm to 5:00 pm are relatively unaffected, with only a few consumers seeking to leave early at 4:00 or 4:30 pm. For those consumers who did not consume during the lunch break, they will often leave by 4:00 pm to do so.

Generally, the data shows that khat consumption habits negatively affects the work culture and economy of a chewer household. In line with this, a study by Taye and Aune indicates khat “consumption negatively affects the working capacity of people” [35]. The authors explained this finding by using some of the variables used in this study, including that khat consumers show up late for work, take frequent rests and spend time-consuming khat. As a result, Taye and Aune [35] find, khat consumers are paid less than non-khat users per day. In addition, they also indicated that consumption of khat has serious social consequences, and consumers spend a high portion of their income to purchase khat [35]. Khat consumption reduces working hours and income of individuals. The potential reduction in working hours can putt the household at risk of job loss and reduced expenditure on basic goods. Although the turnover rate of consumers and non-consumers requires further study, it is possible to gauge that the typical khat consumer work culture makes it challenging to retain permanent, full-time positions. Although this study only offers implications on the broader economy, it is likely that the accumulation of these practices at the societal level reduces the overall productivity of the broader economy, which indirectly can also affect the nation in general including non-consumer households.

## Conclusions

We are cognizant that many people and stakeholders who were not included in this study benefit from khat cultivation, production, and taxation. Farmers cultivating khat obtain higher prices for the sale of khat when compared to other alternative commodities. Multiple levels of government obtain significant tax revenues via the khat trade. We have not excluded these aspects of khat, rather this study contributed evidence regarding the impact of khat consumption habit on the household and work, largely exploring financial and time aspects. Further study is required to address other components of khat cultivation, production, sale, and taxation. Moreover, although this study has presented the linkages between khat consumption habits and the economy of a household, these are correlations and further study is required to determine causality. It is hoped that this study provides direction for future research.

Khat has been chewed for a long time in Harar city and its effects are clearest in this socio-cultural setting as compared to other parts of Ethiopia because of its long history of cultivation and consumption. Although there were only slight differences between consumer and non-consumer households' monthly income, there were great differences regarding their expenditure. Consumer families had additional financial burdens related to khat consumption. These burdens pushed some households to fall into the cycle of borrowing, selling home materials and using money reserved for food. This additional financial burden appears to negatively affect saving practices. A significant amount of time is spent on khat-consuming related activities, and this negatively affects work and work culture. Khat consumers go to work late and leave early. Consumers spent daily an average of 3.75 of their time and 75.8 ETB on a single khat consumption session.

The culture of khat consumption developed over a long period of time and cannot be changed overnight. As Cochrane and Girma [19] note, there is no easy way to address the challenges emanating from khat, while also balancing the benefits some draw from it. Understanding the complexities will help decision-makers in grappling with a complex and multidimensional issue. At this point, the lives and livelihoods of millions of people in Ethiopia depend on khat and khat consumption [5]. One potential avenue for redirecting the industry, while protecting farmers, is to fund research on the potential medical uses of khat, so that the commodity can retain its high value for farmers and also be regulated. For example, there are emerging medical uses of cannabis, alongside varying degrees of regulation. There is also a need, and indeed demand, for health institutions to establish rehabilitation services (as has been done at Mekelle University), particularly for those who say, ‘life for me is chewing’. While the issues are complex and multidimensional, there appear to be actions that can be taken, based upon available evidence and policy experience. This study reinforces the need for regulation and highlights entry-points where this might take place, such as workplace-based regulations and supports to reduce khat consumption habits.

## Supporting information

S1 Table Participants’ profile. (DOCX)

S2 Table Consumer household’s condition and dependence on Khat. (DOCX)

S3 Table Indicators of economic conditions of consumers and non-consumers. (DOCX)

S4 Table Additional burdens of khat consuming households. (DOCX)
