# Supplementary material for: An assessment of khat consumption habit and its linkage to household economies and work culture: The case of Harar city
Source: PLoS One. 2019 Nov 5;14(11):e0224606. doi: 10.1371/journal.pone.0224606 (PMC6830813; doi:10.1371/journal.pone.0224606)
Supplement: S1 Table — (DOCX) [file pone.0224606.s001.docx]

**S1 Table. Participants’ Profile**

| **Profile of Respondents** | | **Consume khat?** | | | |
| --- | --- | --- | --- | --- | --- |
|  |  | **Yes** | | **No** | |
|  |  | **Frequency** | **%** | **Frequency** | **%** |
| Age | >50  40-50  30-39  19-29 | 18  39  69  75 | 9.0%  19.4%  34.3%  37.3% | 33  54  87  23 | 16.8%  27.4%  44.2%  11.7% |
|  | Mean Age | 35.8 | | 39.6 | |
| Sex | Male  Female | 159  42 | 79.1%  20.9% | 108  89 | 54.8%  45.2% |
| Educational Status | Grade 12+ and university graduates  Grade 8-12  Grade 1-7  Not educated | 93  63  27  18 | 46.3%  31.3%  13.4%  9.0% | 72  71  42  12 | 36.5%  36.0%  21.3%  6.1% |
