# Supplementary material for: An assessment of khat consumption habit and its linkage to household economies and work culture: The case of Harar city
Source: PLoS One. 2019 Nov 5;14(11):e0224606. doi: 10.1371/journal.pone.0224606 (PMC6830813; doi:10.1371/journal.pone.0224606)
Supplement: S2 Table — (DOCX) [file pone.0224606.s002.docx]

**S2 Table. Consumer Household’s Condition and Dependence on Khat**

| **Consumer’s Condition** | | **Frequency (Total 201)** | **%** |
| --- | --- | --- | --- |
| Who consumes khat in the family? | father  mother and father  son/daughter  other relatives  the whole family | 113  36  13  8  31 | 56.2%  17.9%  6.5%  4.0%  15.4% |
| For how long you consumed? | >15 years  10-15 years  5-9 years  1-4 years | 60  48  48  45 | 29.9%  23.9%  23.9%  22.4% |
|  | Mean | 12.1 years | |
| Why do you consume khat? | to pray (for worship)  to get energy for work  to have better social interaction  for entertainment  to get good mood for reading | 0  129  15  48  9 | 0.0%  64.2%  7.5%  23.9%  4.5% |
| How often do you consume khat? | the whole day  three times a day  two times a day  once a day  three times a week  two times a week | 4  6  23  99  32  37 | 1.9%  2.9%  11.4%  49.2%  15.9%  18.4% |
| On average how long do you stay normally without consuming? If you do not find khat for a various reason | a day  two days  three days  four days  a week  a month | 12  66  45  21  57  0 | 6.0%  32.8%  22.4%  10.4%  28.4%  0.0% |
| What happens if you do not consume khat as per your schedule? | cannot work well  not treat others well  not happy  isolate myself  fight with others  nothing happens | 96  3  78  3  3  18 | 47.8%  1.5%  38.8%  1.5%  1.5%  9.0% |
