# Supplementary material for: An assessment of khat consumption habit and its linkage to household economies and work culture: The case of Harar city
Source: PLoS One. 2019 Nov 5;14(11):e0224606. doi: 10.1371/journal.pone.0224606 (PMC6830813; doi:10.1371/journal.pone.0224606)
Supplement: S3 Table — (DOCX) [file pone.0224606.s003.docx]

**S3 Table. Indicators of Economic Conditions of Consumers and Non-consumers**

| Variables | | | **Consume khat?** | | | |
| --- | --- | --- | --- | --- | --- | --- |
|  |  |  | **Yes** | | **No** | |
|  |  |  | **Freq.** | **%** | **Freq.** | **%** |
| Employment | employed | | 102 | 50.7% | 141 | 71.6% |
|  | underemployed | | 54 | 26.9% | 36 | 18.3% |
|  | unemployed | | 45 | 22.4% | 20 | 10.2% |
| Monthly income | | >3000 ETB  2000-3000 ETB  1000-1999 ETB  500-999 ETB  <500 ETB | 45  45  48  45  18 | 22.4%  22.4%  23.9%  22.4%  9.0% | 57  45  33  42  20 | 28.9%  22.8%  16.8%  21.3%  10.2% |
|  |  | Mean | 2064 ETB | | 2103 ETB | |
| Daily expenditure for basic family needs (like food and water) | | >150 ETB  50-150 ETB  20-49 ETB  <20 ETB | 24  69  63  45 | 11.9%  34.3%  31.3%  22.4% | 21  90  42  44 | 10.7%  45.7%  21.3%  22.3% |
|  |  | Mean | 52 ETB | | 63 ETB | |
