# Supplementary material for: An assessment of khat consumption habit and its linkage to household economies and work culture: The case of Harar city
Source: PLoS One. 2019 Nov 5;14(11):e0224606. doi: 10.1371/journal.pone.0224606 (PMC6830813; doi:10.1371/journal.pone.0224606)
Supplement: S4 Table — (DOCX) [file pone.0224606.s004.docx]

**S4 Table. Additional Burdens of Khat Consuming Households**

| **Variables** | | **Frequency** | **%** |
| --- | --- | --- | --- |
| After *barcha,* drinking is___ | essential  desirable  not necessary | 21  45  135 | 10.4%  22.4%  67.2% |
| Use of other substances | yes  no | 87  114 | 43.3%  56.7% |
| Total expenditure on a single khat consumption session | >150 ETB  50-150 ETB  20-49 ETB  <20 ETB | 45  48  54  54 | 22.4%  23.9%  26.9%  26.9% |
|  | Mean | 75.8 | |
| Time spent on one consumption ceremony | >5 hours  3-5 hours  1-2 hours | 36  123  42 | 17.9%  61.2%  20.9% |
|  | Mean | 3.75 | |
